# Mechanical and Thermal Characteristics of Cement Composites Containing PEDOT:PSS and Amorphous Metallic Fibers

**DOI:** 10.3390/ma18194486

**Published:** 2025-09-26

**Authors:** Se-Jin Choi, Jeong-Yeon Park, Min-Jeong Kim, Jae-In Lee

**Affiliations:** Department of Architectural Engineering, Wonkwang University, 460 Iksan-daero, Iksan 54538, Republic of Korea; csj2378@wku.ac.kr (S.-J.C.); jkite7490@naver.com (J.-Y.P.); mjcon@hdec.co.kr (M.-J.K.)

**Keywords:** PEDOT:PSS, amorphous metallic fiber, conductive, mortar, thermal properties

## Abstract

Poly(3,4-ethylenedioxythiophene):poly(styrenesulfonate) (PEDOT:PSS) is a conductive polymer that has attracted significant attention in various industries. However, studies on the application of PEDOT:PSS in cement composites are scarce. The thermal performance and mechanical properties of conductive cement composites manufactured using amorphous metallic fibers (AFs), reinforcing fibers with excellent conductivity in concrete, and the conductive polymer PEDOT:PSS in various ratios are investigated in this study. When only PEDOT:PSS and a combination of AFs and PEDOT:PSS are used, the splitting tensile strength of the composite at 28 d increases by 15.4% and 38.5%, respectively, compared with that of the plain sample (without PEDOT:PSS and AFs). Additionally, the simultaneous incorporation of PEDOT:PSS and AFs significantly reduces the brittleness of cement composites. The heat-generation performance shows minimal changes when only PEDOT:PSS is used; however, when 0.6% PEDOT:PSS and AFs are used together, a temperature increase rate of 182% is observed, which is 138% better than that of the plain sample. In scanning electron microscopy–energy-dispersive X-ray spectroscopy analysis, spherical hydrates, likely comprising PEDOT, are observed in samples incorporated with only PEDOT:PSS; samples incorporated with PEDOT:PSS and AFs show hydrates with a clearer shape than those observed in the plain sample. This study is expected to open new frontiers in the design and development of high-performance additive-incorporated cement composites with unique properties for specific applications.

## 1. Introduction

Conductive cement composites have attracted considerable attention for their self-sensing capabilities, which enable them to detect the interior of structures, and for their potential to mitigate accidents caused by dirty roads at subzero temperatures [1,2,3]. Roads, particularly in winter, cause traffic delays and black ice, leading to traffic accidents [4,5]. Various studies have attempted to address these issues and mitigate road-icing problems by imparting electrical conductivity to cement composites. Owing to the inherently low electrical conductivity of cement composites, most studies have focused on improving their conductivity by incorporating conductive materials [6,7,8]. The conductive materials incorporated into electrically conductive cement composites are primarily carbon-based, such as carbon nanotubes (CNTs), carbon black, and carbon fiber (CF) [9]. CNTs are expensive and might agglomerate if dispersed non-uniformly in cement composites, potentially affecting their performance characteristics [10]. Similarly, CFs are associated with certain issues, such as uneven dispersion within cement composites [11] and weak bonding with the cement matrix owing to smooth surfaces [12]. The recently reported conductive polymer poly(3,4-ethylenedioxythiophene):poly(styrenesulfonate) (PEDOT:PSS) comprises positively charged PEDOT and the negatively charged surfactant PSS [13,14,15]. PEDOT:PSS exhibits several advantageous properties, such as low cost and high thermal stability, and is being increasingly used in various industries [16,17]. When combined with conductive materials, the electrical conductivity of PEDOT:PSS is enhanced further [18,19]. In addition, owing to the presence of PSS, PEDOT:PSS can improve the dispersibility of low-dispersibility materials, such as CNTs, in cement composites. When used in combination with PEDOT:PSS, the dispersibility of CNTs, as well as their electrical conductivity, is improved significantly [18,19].

Another method for manufacturing conductive cement composites involves the incorporation of metal- and carbon-based reinforcing fibers into concrete. On incorporating these materials into concrete, the fibers dispersed within the matrix form conductive pathways, thereby enhancing the electrical conductivity of the cement composite [20,21,22]. The types of fibers used in this process include CFs, amorphous metallic fibers (AFs), and those comprising steel.

Notably, steel fibers dispersed within a cement matrix may undergo corrosion, whereas CFs may undergo weak bonding with the matrix [12]. In contrast, AFs show excellent corrosion resistance because they are non-crystalline materials; moreover, their surfaces develop a rough texture during the manufacturing process, enabling strong bonding with the cement matrix [23,24]. Notably, the incorporation of AFs improves the electrical performance of cement composites significantly [25]. The literature indicates that the combined use of the highly conductive PEDOT:PSS and AFs, which exhibit excellent corrosion resistance and strong bonding with the cement matrix, is likely to enhance both the electrical conductivity and mechanical performance of cement composites. However, studies on the application of PEDOT:PSS in cement composites are scarce, and there are no reports on the use of PEDOT:PSS in combination with AFs.

This study investigates the flowability as well as the mechanical and thermal performance of conductive cement composites containing PEDOT:PSS and AFs. In this study, cement composites were prepared using different concentrations of PEDOT:PSS (0%, 0.3%, 0.6%, and 0.9% by weight of cement) and AFs (0 and 10 kg/m). The flowability, hydration heat, mechanical properties, heat-generation performance, and scanning electron microscopy–energy-dispersive X-ray spectroscopy (SEM–EDS) results of these composites were analyzed.

## 2. Materials and Methods

### 2.1. Materials

Type 1 ordinary Portland cement (Sampyo Cement, Seoul, Republic of Korea) with a density and Blaine fineness of 3.13 g/cm^3^ and 3820 cm^2^/g, respectively, and a fine aggregate comprising crushed sand, with a density and fineness modulus of 2.60 g/cm^3^ and 2.45, respectively, was used in this study.

In addition, AFs with a density of 7.2 g/cm^3^ and length of 15 mm (Saint-Gobain SEVA, France) were used. Because the fiber length can influence the mechanical properties of cement composites [26], the AF length used in this study was determined based on the literature [27] and preliminary experiments. In addition, the fiber content was set to 0 or 10 kg/m^3^ because preliminary experiments indicated that a fiber content exceeding 10 kg/m^3^ adversely affected workability. An aqueous solution of PEDOT:PSS (Sooyangchemtec, Yesan, Republic of Korea) with a solid content of 0.8–1.1% and pH of 1.5–2.5 was used in this study; different amounts of PEDOT:PSS (0%, 0.3%, 0.6%, or 0.9% by weight of cement) was added to cement. The properties of the AFs and PEDOT:PSS used in this study are listed in Table 1 and Table 2.

**Figure 1:**
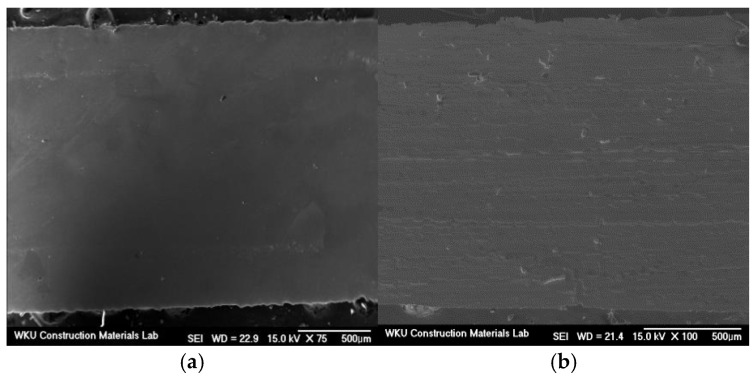
The (**a**) smooth and (**b**) rough surface regions of the amorphous metallic fibers.

**Figure 2:**
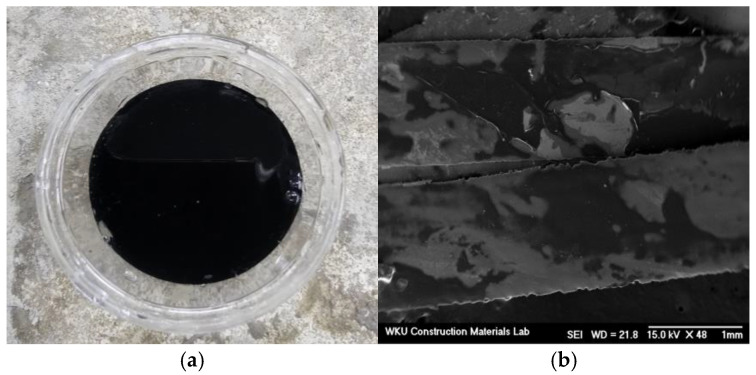
(**a**) The PEDOS:PSS solution, and (**b**) scanning electron microscopy (SEM) image of PEDOT:PSS coated on amorphous metallic fibers.

A SEM image of the AFs, which exhibit two types of surface morphologies (smooth and rough) is shown in Figure 1. PEDOT:PSS exists as an aqueous black solution, as shown in Figure 2. Owing to the nature of SEM, this technique cannot be used to record images of materials in aqueous solution. Therefore, SEM images were recorded after drying AFs moistened with the aqueous solution.

**Figure 3:**
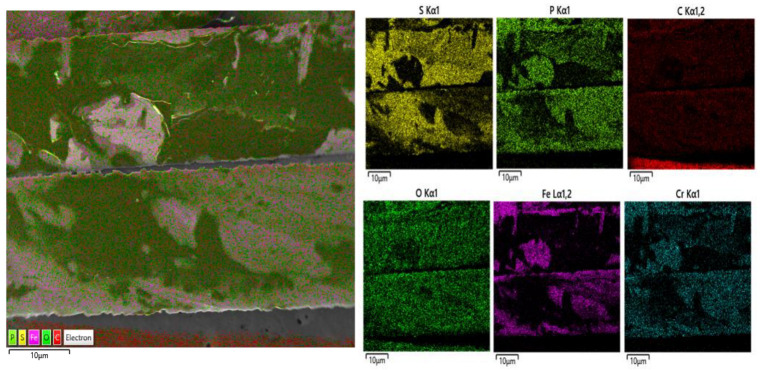
Energy-dispersive X-ray spectroscopy (EDS) mapping results.

The EDS mapping results of AFs coated with PEDOT:PSS are shown in Figure 3. The AFs are composed of Fe, Cr, and a small amount of P. The dark areas in the image indicate regions where PEDOT:PSS is applied, primarily showing the S components detected in PEDOT:PSS [28].

### 2.2. Mixing Proportions and Specimen Synthesis

**Figure 4:**
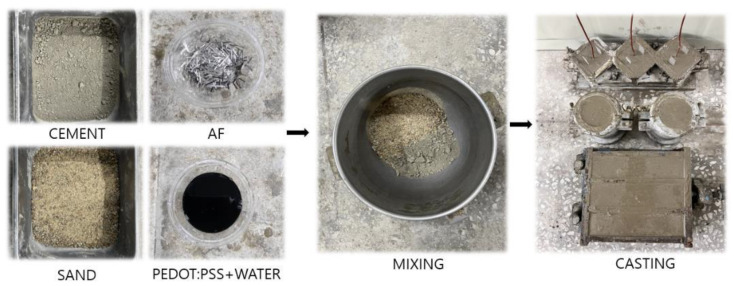
The test-sample production process.

Table 3 lists the experimental mixture parameters used in this study. The water/cement ratio was fixed at 0.5 in all experiments. Plain samples without PEDOT:PSS and AFs, samples with PEDOT:PSS, and samples with both PEDOT:PSS and AF were prepared. Cubic specimens (50 mm × 50 mm × 50 mm) were fabricated to evaluate the compressive strength and heat-generation performance of the cement composites containing PEDOT:PSS and AFs, as shown in Figure 4. Additionally, prismatic specimens (40 mm × 40 mm × 160 mm) were fabricated for flexural strength testing, and a cylindrical specimen with dimensions of Ø50 × 100 mm was manufactured to measure the splitting tensile strength. For specimens without PEDOT:PSS and AF, fine aggregate and cement were dry-mixed for 30 s. Subsequently, mixing water was added, and the mixture was further blended for an additional 90 s, resulting in a total mixing time of 120 s. In the case of specimens containing PEDOT:PSS, the PEDOT:PSS was combined with the mixing water, and the total mixing time also remained at 120 s. For specimens incorporating AF, after the initial dry-mixing, mixing water was introduced and blended for 30 s to ensure adequate workability of the mortar prior to the addition of AF. The total mixing time for all specimens was consistently maintained at 120 s. For microstructural analysis using SEM and EDS, core fragments from the specimens were collected after 28 d compressive strength testing. Approximately 24 h after fabrication, the specimens were demolded and cured in water at 20 °C to ensure adequate aging.

The mortar flow and compressive strength were measured according to the test methods specified in KS L 5111 [29] and KS L 5105 [30]. The microhydration heat was measured using a semi-adiabatic calorimeter (Calmetrix, F-Cal8000, Boston, MA, USA) in accordance with ASTM C 1753 [31], and the splitting tensile strength was measured in accordance with KS F 2423 [32]. The flexural strength was measured according to KS F 2408 [33], and the microstructure was analyzed using SEM (AIS1800C, Seron, Uiwang-si, Republic of Korea) and EDS (OXFORD INSTRUMENTS, Xplore, Abingdon, UK).

**Figure 5:**
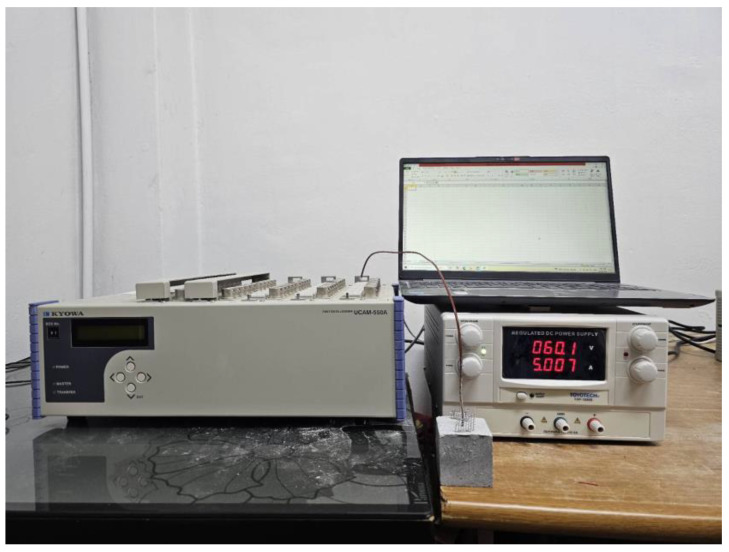
Thermal performance test setup.

For heat-generation performance testing using the setup shown in Figure 5, a 50 × 50 × 50 cubic test specimen was fabricated using a previously published method [21,25]. A K-TYPE thermocouple was inserted into the center of the sample to monitor its internal temperature change over time with voltage application. Electrodes comprising stainless steel were inserted at 40 mm intervals on both sides of the test specimen. A voltage of 60 V was applied, and the temperature change was automatically monitored using a data logger.

## 3. Results and Discussion

### 3.1. Mortar Flow

**Figure 6:**
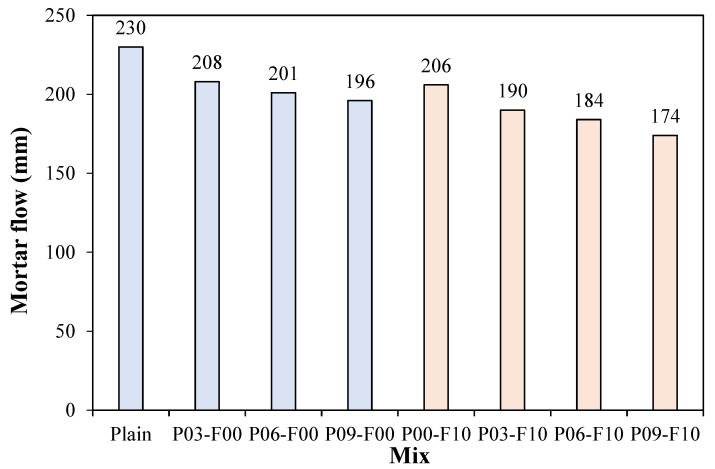
Changes in mortar flow for the prepared specimens.

Changes in mortar flow for the fabricated samples are shown in Figure 6. The mortar flow is the highest at ~230 mm (when PEDOT:PSS is not mixed in) and decreases with increasing PEDOT:PSS content. The mortar flow of the sample containing 0.3% PEDOT:PSS decreases by ~9.6%, while that of the sample containing 0.9% PEDOT:PSS decreases by ~14.8%, resulting in a flow of ~196 mm. When AFs and PEDOT:PSS are used together, the mortar flow exhibits a decreasing trend, similar to the trend observed with only PEDOT:PSS. When 0.9% PEDOT:PSS and AF (10 kg/m^3^) are used, the mortar flow (~174 mm) is ~11.3% lower than that of the P09-F00 sample with the same PEDOT:PSS content. As PEDOT:PSS contains the surfactant PSS, the mortar flow is expected to increase with increasing PEDOT:PSS content. However, the opposite trend is observed in this study. The observed reduction in mortar flow on adding PEDOT:PSS can be attributed to two reasons. First, PEDOT:PSS absorbs moisture [34]; second, when exposed to moisture, the chains between PEDOT and PSS weaken [35]. The weakening of PEDOT:PSS bonds may cause PEDOT and PSS to separate, resulting in the aggregation of hydrophobic PEDOT molecules [36,37], which may reduce the mortar flow. Furthermore, the significant reduction in mortar fluidity on incorporating AFs can be attributed to moisture loss, which contributes toward fluidity owing to the introduction of dry fibers, and the dispersion of fibers, which partially interferes with the mortar flow.

### 3.2. Heat of Hydration

**Figure 7:**
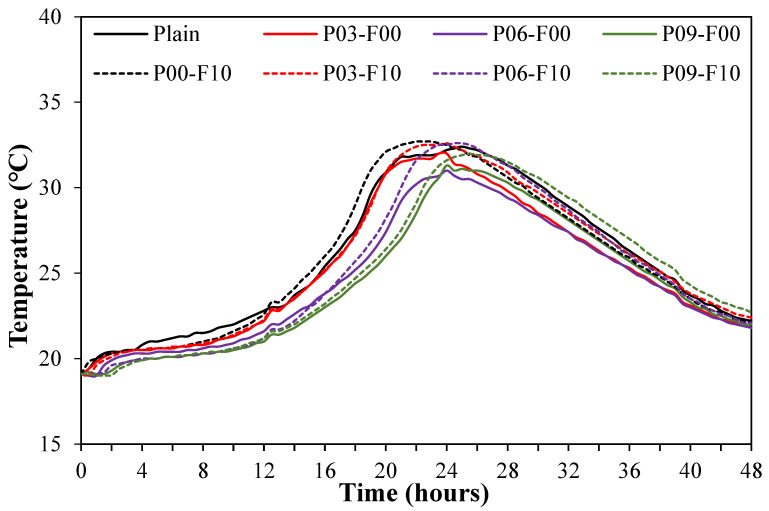
Variation in the microhydration heat with time.

Changes in the hydration heat of the mortar mixed with PEDOT:PSS and AFs are shown in Figure 7; the maximum hydration temperature decreases with increasing PEDOT:PSS content.

Specifically, the maximum hydration temperature of the plain sample (without PEDOT:PSS and AFs) is 32.4 °C, while that of the sample containing only PEDOT:PSS lies within 31.0–32.0 °C. Interestingly, both samples attain the maximum hydration temperature within a similar time (at ~23–24 h); the plain sample attains it at ~24 h. The slight reduction in maximum hydration temperature on adding PEDOT:PSS can be attributed to the weakening of the bond between PEDOT and PSS, consistent with the results shown in Figure 6 (mortar flow) and the reduction in the contact area between the cement pastes due to the aggregation of the separated hydrophobic PEDOT, resulting in delayed hydration reactions. Overall, the maximum temperature onset time is similar for samples with and without PEDOT:PSS, suggesting that the addition of PEDOT:PSS influences the hydration reaction rate of cement negligibly. When PEDOT:PSS and AFs are combined, the maximum hydration temperature decreases slightly with increasing PEDOT:PSS content. When only AFs are used, the maximum hydration temperature is ~32.7 °C, which is the highest maximum hydration temperature observed across samples. In contrast, when PEDOT:PSS is used in combination with AFs, the maximum hydration temperature lies within 32.0–32.6 °C, which is similar to (or slightly higher than) the maximum hydration temperature of the plain sample; in addition, the time required to attain the maximum temperature is 22–25 h, with a difference of ~3 h. When only AFs are used, this time is reduced to 22 h, which is the shortest time required to attain the maximum hydration temperature, whereas when PEDOT:PSS and AFs are used together, this time is ~24–25 h, which is similar to the time observed for the plain sample and the sample using only PEDOT:PSS. However, the maximum temperature when AFs are used is 5.5% higher than when only PEDOT:PSS is used. This can be attributed to the excellent thermal conductivity of AFs dispersed within the matrix, which ensures that the heat generated by the cement hydration reaction is spread uniformly throughout the system [38].

### 3.3. Compressive Strength

**Figure 8:**
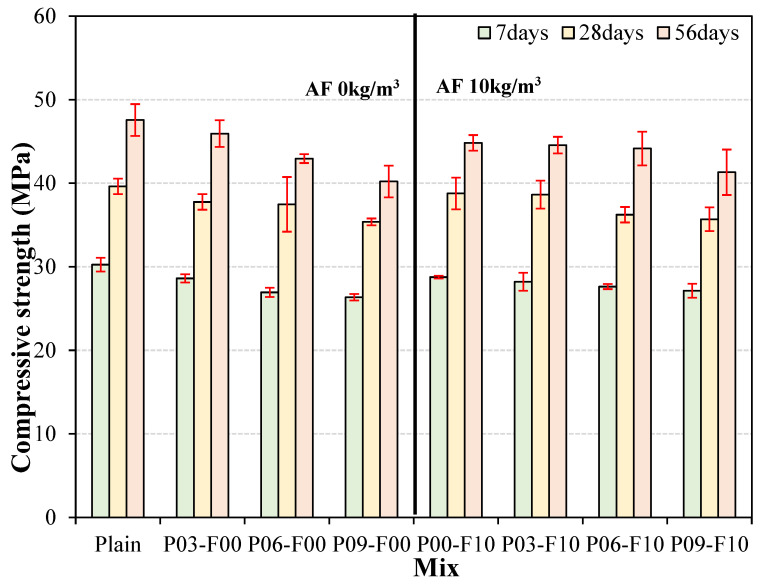
Variation in compressive strength with age for systems containing different amounts of PEDOT.

Changes in the compressive strength of systems containing different amounts of PEDOT with age are shown in Figure 8. The compressive strength of all samples increases continuously with increasing age. On PEDOT:PSS addition, the compressive strength decreases slightly on increasing the PEDOT:PSS addition rate. Similarly, when PEDOT:PSS and AFs are used together, the compressive strength decreases on increasing the PEDOT:PSS addition rate. This phenomenon can be attributed to the weakening of the bonds in PEDOT:PSS upon contact with moisture, which leads to the aggregation of the PEDOT separated from PSS and the formation of numerous pores around the aggregates, thereby reducing the homogeneity of the matrix [39]. Additionally, when AFs are co-blended, the reduction in compressive strength can be attributed to pores formed by fiber blending, as reported in previous studies [40,41]. Notably, the compressive strength of the AF-incorporated samples at 56 d of curing is similar to (or slightly higher than) that of samples incorporated with only PEDOT:PSS. As the purpose of using reinforcing fibers involves improving the bending and tensile performance (rather than the compressive strength) of the system, AF incorporation is not expected to significantly influence the compressive strength of PEDOT:PSS-based cement composites. Furthermore, at 7 days of age, the deviation among test specimens ranged from approximately 0.6% to 3.8%, indicating minimal variation in quality between samples. At 28 days of age, this deviation increased slightly, ranging from approximately 1.2% to 8.7%, before decreasing somewhat to approximately 1.2% to 6.6% at 56 days of age. No discernible trend was observed in sample deviation with the incorporation of PEDOT:PSS and AFs. Across all ages, the maximum deviation remained within 10%, which is indicative of good quality.

**Figure 9:**
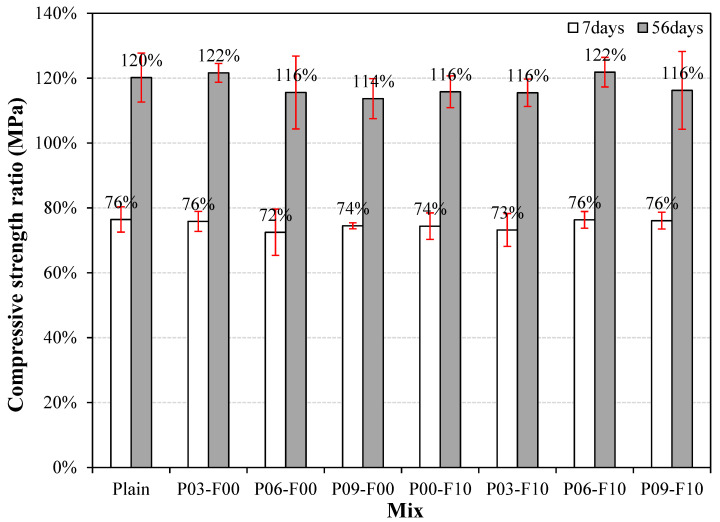
Development of the compressive strength ratio for different samples.

The compressive-strength development rate based on the compressive strength at 28 d is shown in Figure 9. The compressive-strength development rate at 7 d is ~72–76%, and when PEDOT:PSS and AFs are mixed, it is similar to or slightly lower than that of the plain sample. Notably, the plain sample shows a development rate of ~120% at 56 d of curing, which increases to ~122% on adding only PEDOT:PSS (0.3%). When AFs are used in combination with PEDOT:PSS, the values range from ~115% to 122%, indicating that some samples show a slightly higher compressive-strength development rate than the plain sample. These results suggest that while the incorporation of PEDOT:PSS may reduce the compressive strength slightly owing to the formation of internal voids, it does not affect the long-term compressive-strength development significantly. Further research is required to assess the long-term performance of the system beyond 56 days. Regarding the variation in compressive strength development rate, similar to compressive strength, it ranged from approximately 0.9 to 5.1% at 7 days, demonstrating excellent quality. Conversely, at 56 days, it reached a maximum of 12.0%, a relatively high figure compared to 7 days. However, a 12% variation rate is still not significant.

### 3.4. Splitting Tensile Strength

**Figure 10:**
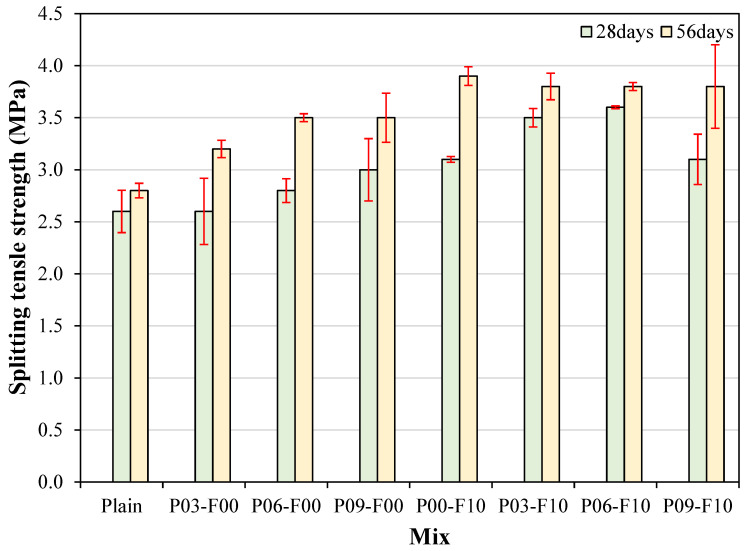
Variation in the splitting tensile strength with age.

The variation in the splitting tensile strength of different samples with age is shown in Figure 10. Among the tested samples, the plain sample exhibits the lowest splitting tensile strength of ~2.6 MPa. The splitting tensile strength of the samples incorporating only PEDOT:PSS measured at 28 d (ranging from ~2.6 to 3.0 MPa) is up to 15.4% higher than that of the plain sample and significantly lower than that of the samples incorporating AFs (which show a splitting tensile strength value within ~3.1–3.6 MPa). The increase in splitting tensile strength on PEDOT:PSS addition can be attributed to the unique properties of PEDOT:PSS, a conductive polymer with an excellent elastic modulus [42]. Therefore, the enhanced splitting tensile strength of the modified cement composites can be attributed to the high elasticity of the incorporated materials, which enables them to resist externally applied tensile forces [43].

The splitting tensile strength of the PEDOT:PSS-incorporated samples increases significantly after 56 d. Specifically, the splitting tensile strength of samples containing only PEDOT:PSS ranges from ~3.2 to 3.5 MPa, representing an increase of up to 25% compared with the plain sample, which shows a strength of ~2.8 MPa.

The 56 d splitting tensile strength of the AF-incorporated samples lies within ~3.8–3.9 MPa, representing an increase of up to 21.9% compared with samples using PEDOT:PSS alone and an increase of ~39.3% compared with the plain sample. The observed enhancement in tensile performance on AF incorporation can be attributed to excellent fiber/matrix interfacial bonding and the inherently high tensile strength of AFs. Notably, samples incorporated with AFs show a significant improvement in tensile performance compared with the plain sample, even when used in conjunction with PEDOT:PSS. The variability of the splitting tensile strength was somewhat greater than that of the compressive strength. Specifically, at 28 days, the minimum was 0.4%, but the maximum was 0.2%. However, at 56 days, the minimum was 1.0%, and the maximum was 10.6%, showing a tendency for the variability to be alleviated compared to 28 days. The variability of the splitting tensile strength also showed good values similar to that of the compressive strength, showing that the incorporation of PEDOT:PSS and AFs did not have a significant effect on the variability.

**Figure 11:**
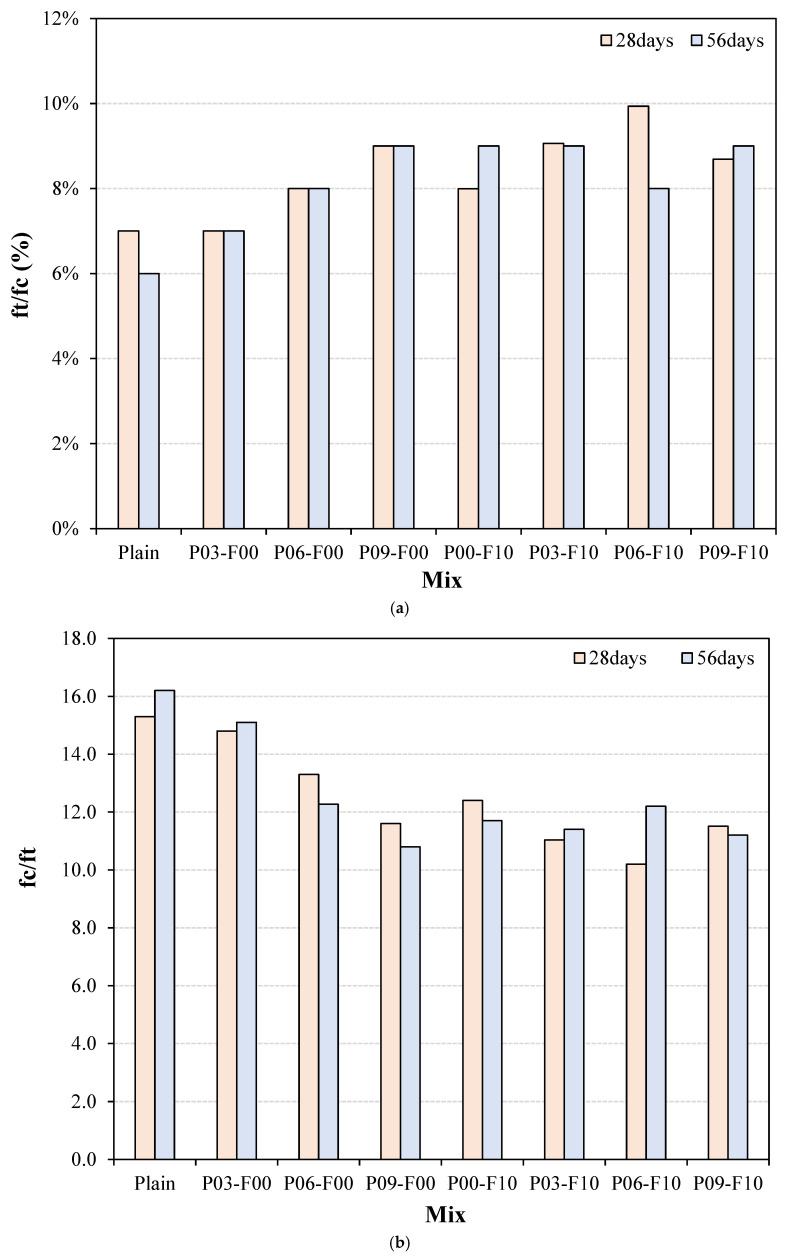
Values of ft/fc and fc/ft for the different samples synthesized in this study. Here, ft represents the splitting tensile strength and fc represents the compressive strength. (**a**) ft/fc; (**b**) fc/ft.

The ratio between the splitting tensile strength (ft) and the compressive strength (fc) for the samples synthesized in this study is shown in Figure 11. According to the literature, the incorporation of reinforcing materials generally leads to an enhancement in the tensile performance of concrete, resulting in a high ft/fc ratio in cementitious composites [44]. As expected, the plain sample shows the lowest 28 d ft/fc value (~7%) among all the tested samples. Samples treated with PEDOT:PSS alone exhibit a ratio of ~7–9%, whereas those treated with both PEDOT:PSS and AFs show a ratio of ~8–10%, indicating an improvement compared with the plain sample. Numerous studies have demonstrated that incorporating fibers can increase the tensile resistance of materials [45,46]. However, no studies have yet explored the use of PEDOT:PSS to enhance the tensile performance of cementitious composites. The results of this study demonstrate that the sole addition of PEDOT:PSS can achieve a higher ft/fc ratio than conventional cementitious composites and generally achieve levels comparable to those achieved with fibers.

The fc/ft ratio is used as an indicator of the brittleness index; a material with a higher brittleness index shows higher brittleness [47]. The 28 d brittleness index of the plain sample (~15.3) increases to ~16.2 at 56 d, which is the highest observed level of brittleness in this study. The brittleness index values of the samples incorporating PEDOT:PSS measured after 28 d range from ~11.6 to 14.8. After 56 d, index values within ~10.8–15.1 are observed, indicating a reduction in brittleness compared with the plain sample. Notably, the brittleness index of the sample containing 0.9% PEDOT:PSS measured after 56 d (~10.8) is ~33.3% lower than that of the plain sample. When PEDOT:PSS is used with AF, the brittleness index at 28 d lies approximately within 10.2–11.5; the P06-F10 sample exhibits the lowest brightness index of 10.2. Notably, the maximum brittleness index of the 56 d test specimens (~12.2) is ~24.7% lower than that of the plain sample. In addition, while a maximum brittleness index of up to ~15.1 is observed when only PEDOT:PSS is added, the maximum brittleness index increases only up to ~12.2 when AFs are added, suggesting the possibility of mitigating brittleness with AF addition.

### 3.5. Flexural Strength

**Figure 12:**
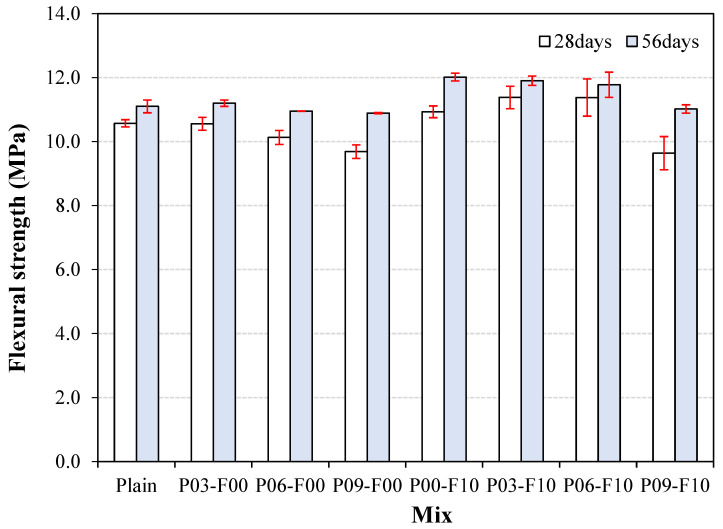
Variation in the flexural strength of the samples.

Changes in the flexural strength of the cement composites mixed with PEDOT:PSS and AFs are shown in Figure 12. The 28 d flexural strength of the plain sample is ~10.6 MPa. In contrast, the flexural strength of samples using PEDOT:PSS lies within 9.7–10.6 MPa; the P09-F00 sample exhibits the lowest flexural strength of ~9.7 MPa. The flexural strength of samples incorporating both PEDOT:PSS and AFs lies approximately within 9.6–11.4 MPa, indicating an improvement of up to 10.5% compared with the plain sample.

The flexural strength of the samples containing PEDOT:PSS are observed to be the lowest for P09-F00 and P09-F10 (9.6 and 9.7 MPa, respectively) with a PEDOT:PSS ratio of 0.9%. This can be attributed to a reduction in matrix homogeneity owing to the non-uniform dispersion of PEDOT separated from weakened PEDOT:PSS. Similarly, with increasing incorporation of PEDOT:PSS, a reduction in flexural strength is observed in the 56 d aged specimens. When only PEDOT:PSS is used, the 56 d flexural strength lies approximately within 10.9–11.2 MPa, which is similar to the flexural strength of the plain sample (~11.1 MPa). Notably, the lowest flexural strength is observed when the incorporated PEDOT:PSS ratio is 0.9%. A similar phenomenon is observed when AFs and PEDOT:PSS are applied simultaneously. Specifically, the flexural strength of the sample incorporated with only AFs (10 kg/m^3^) is ~12.0 MPa, which is ~8.1% higher than the flexural strength of the plain sample. However, when mixed with PEDOT:PSS (0.9%), the flexural strength of the sample (~11.0 MPa) is similar to that of the plain sample.

### 3.6. Thermal Properties

**Figure 13:**
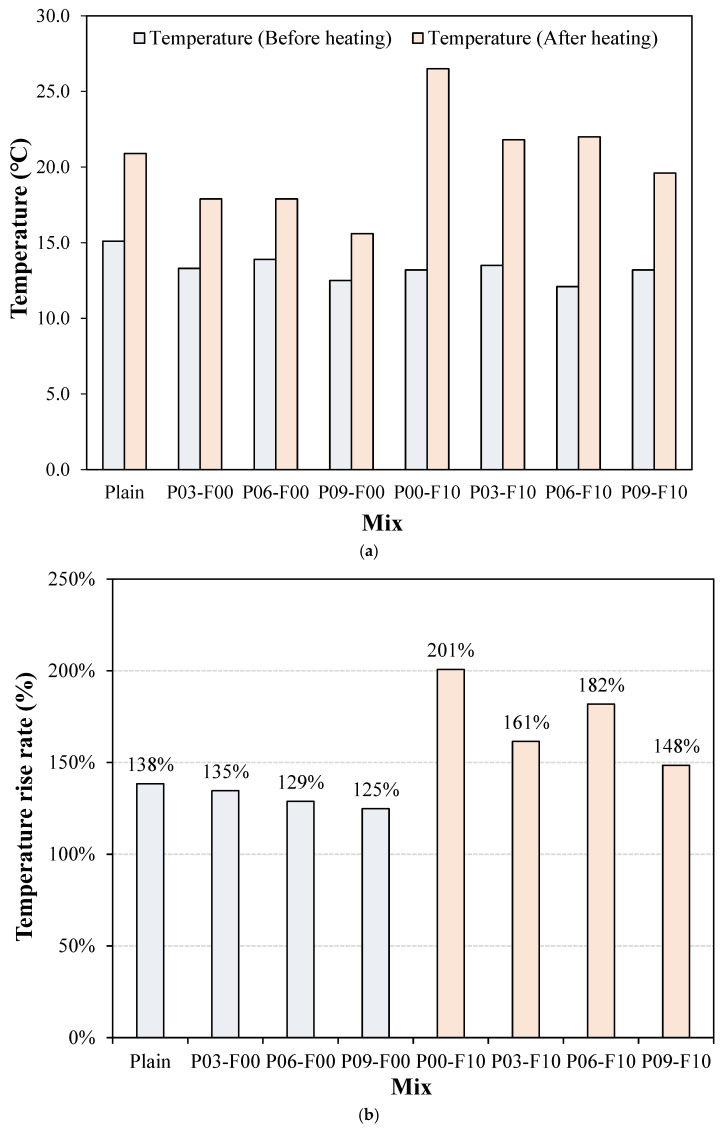
Thermal properties of the synthesized samples. (**a**) Temperature variation with applied voltage; (**b**) Rate of temperature increase with applied voltage.

**Figure 14:**
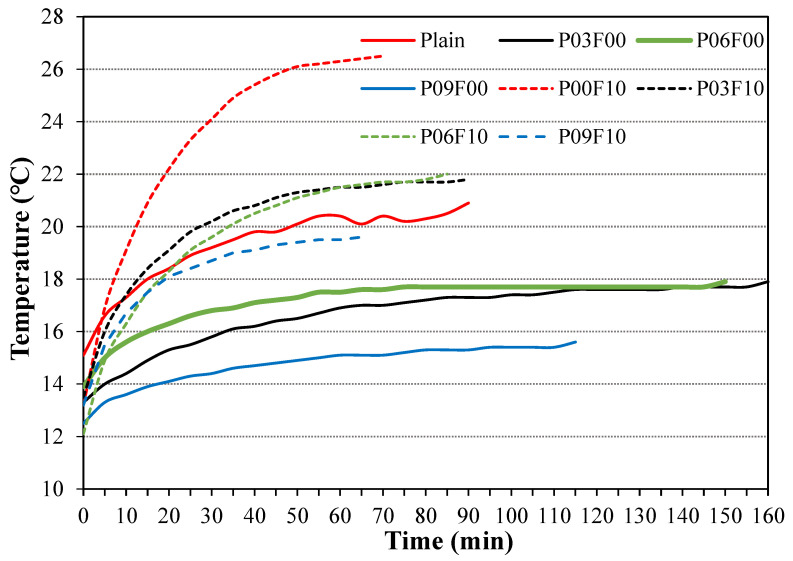
Temperature changes in the synthesized samples over time.

The heat-generation properties of the samples incorporated with PEDOT:PSS and AFs are shown in Figure 13, and the temperature variation in the samples, indicating the point at which each sample exhibits its maximum temperature, is shown in Figure 14. The temperature of the PEDOT:PSS-incorporated sample is lower than that of the plain sample following voltage application (Figure 13). On voltage application, the initial temperature of the plain sample (~15 °C) increases (by ~138%) to ~20.9 °C. Samples using only PEDOT:PSS exhibit a temperature increase of ~125–135%, which is somewhat lower than that of the plain sample. Therefore, despite the ability of the conductive polymer PEDOT:PSS to effectively enhance electrical conductivity, it shows negligible impact on the thermal performance of cement composites in this study. The negligible improvement in heat-generation performance observed on incorporating a conductive polymer is likely attributable to two factors: inadequate homogeneous dispersion resulting from the separation of PEDOT:PSS and the aggregation of the hydrophobic PEDOT. Although a conductive network is necessary for conductivity enhancement, the aggregation phenomenon may induce porosity, leading to a reduction in internal homogeneity [36,37]. Conversely, the maximum temperature of the sample with AFs is ~26.5 °C, which is ~5.7 °C higher than that of the plain sample; this represents an approximate increase of 201% in temperature compared with the pre-voltage-application state. Additionally, when PEDOT:PSS is used in combination with AFs, the rate of temperature increase lies approximately within 148–161%, which is an increase of ~10–23% compared with that of the plain sample.

The time required by the synthesized samples to show the maximum temperature is shown in Figure 14. The AF-incorporated samples show a significantly higher maximum temperature than samples without AFs, which they attain faster than the samples without AFs. This phenomenon likely occurs because the main component in AFs is Fe (Figure 3), which (similar to other metallic materials) shows high thermal conductivity [48].

### 3.7. Microstructural Analysis

**Figure 15:**
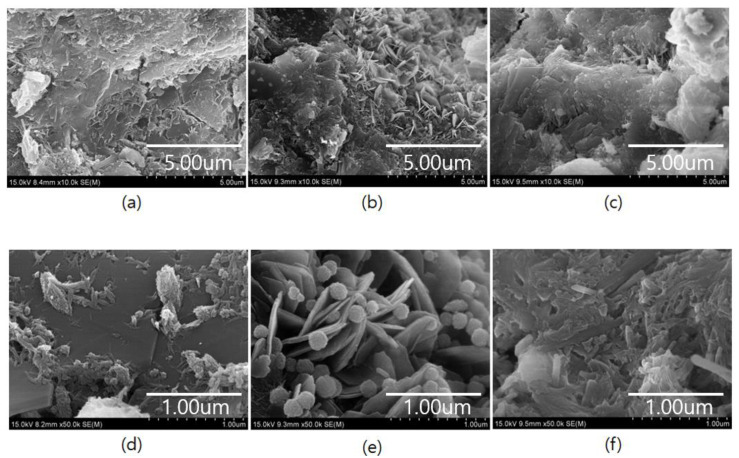
Microstructural analysis (SEM) results for (**a**) the plain sample (×10,000), (**b**) P06-F00 (×10,000), (**c**) P06-F10 (×10,000), (**d**) the plain sample (×50,000), (**e**) P06-F00 (×50,000), and (**f**) P06-F10 (×50,000).

SEM images of the 28 d aged samples are shown in Figure 15. The plain sample, P06-F00, and P06-F10, which show the highest splitting tensile strengths at 28 d, were analyzed by SEM. The matrix of the PEDOT:PSS-incorporated samples exhibit more hydrate formation than the plain sample (Figure 15). In particular, P06-F00 contains dense needle-shaped ettringite, whereas sample P06-F10 contains a matrix surrounded by hydration products. SEM images revealed that the hydrates observed in the samples containing PEDOT:PSS and AF were generally those observed in typical cement composites, such as Ca(OH)_2_, ettringite, and CSH gel. Furthermore, numerous spherical particles, consistent with the morphology of PEDOT:PSS in the literature, are observed in the SEM image of P06-F00, which contains 0.6% PEDOT:PSS (Figure 15e) [49].

The improved tensile properties observed in the samples synthesized with PEDOT:PSS and AFs can be attributed to the inherent flexibility of these materials and their potential to promote hydrate formation. These factors likely contribute toward the higher splitting tensile strength measured at 28 d compared with that of the plain sample. Additionally, the compressive strength of samples incorporating PEDOT:PSS was lower than that of the plain samples. However, the evaluation of compressive strength rate showed that the development rate at 56 days was higher for some samples incorporating PEDOT:PSS compared to the plain samples. This suggests that while separated PEDOT may reduce homogeneity and result in slightly lower initial compressive strength, it does not negatively impact long-term hydration product formation. Similarly, although the compressive strength at 28 days for samples incorporating PEDOT:PSS was lower than that of the plain sample, numerous hydration products affecting strength development were observed.

## 4. Conclusions

The principal findings of this study can be summarized as follows:

1. The heat of hydration decreases with increasing PEDOT:PSS content, likely owing to the aggregation of the hydrophobic PEDOT, which reduces the contact area between the cement paste particles. However, AF incorporation increases the maximum hydration temperature, likely owing to its excellent thermal conductivity.

2. The compressive strength of cement composites incorporating PEDOT:PSS and AFs is only slightly lower than that of the plain sample. Furthermore, at 56 d of curing, some samples incorporating both PEDOT:PSS and AFs exhibit a marginally higher compressive strength than samples incorporating only PEDOT:PSS. According to compressive-strength development rate analysis, the use of PEDOT:PSS and AFs may lead to a slight reduction in compressive strength with negligible long-term impact.

3. Samples treated with PEDOT:PSS exhibit higher splitting tensile strengths than the plain sample. Notably, AF incorporation results in an ~38.5% increase in the splitting tensile strength of plain samples.

4. After 28 d of curing, the flexural strength of samples incorporated with PEDOT:PSS is similar to that of the plain sample. At 56 d, some samples incorporated with PEDOT:PSS show a higher flexural strength than the plain sample. When both AFs and PEDOT:PSS are used, the flexural strength improves further. Therefore, the addition of PEDOT:PSS (with excellent elasticity) and AFs (which can delay crack propagation) can improve the tensile properties of cement composites.

5. The thermal performance of samples incorporated with only PEDOT:PSS is slightly lower than that of the plain sample, likely due to the aggregation of PEDOT separated from PSS, which hinders the formation of conductive paths. However, samples incorporated with AFs exhibit a significantly better performance than the plain sample. This performance improvement can be mainly attributed to the formation of conductive paths by the Fe-based AFs.

This study is expected to guide future research on the design of high-performance cement composites. Further research is required to determine the optimal PEDOT:PSS incorporation method and concentration for maximally enhancing the thermal properties of cement composites.

## Figures and Tables

**Table 1:** Physical properties of amorphous metallic fibers used in this study.

Type	Density (g/cm^3^)	Tensile Strength (N/mm^2^)	Length (mm)
Amorphous metallic fiber	7.2	1400	15

**Table 2:** Physical properties of the PEDOT:PSS used in this study.

Type	Solid Content (%)	pH (-)	Conductivity (S/cm)
PEDOT:PSS	0.8–1.1	1.5–2.5	230

**Table 3:** Mixing proportions of the mortars used in this study.

Type	PEDOT:PSS (C*wt%)	PEDOT:PSS (g)	AF (g)	Water (g)	Cement (g)	Sand (g)
Cubic (50 × 50 × 50 mm) Cylindrical (Ø50 × 100 mm) Prismatic (40 × 40 × 160 mm)	0	0	0	856	1712	4476
	0.3	5.1				
	0.6	10.2				
	0.9	15.3				
	0	0	26.7			
	0.3	5.1				
	0.6	10.2				
	0.9	15.3				

## Data Availability

The original contributions presented in this study are included in the article. Further inquiries can be directed to the corresponding author.

## Abbreviations

The following abbreviations are used in this manuscript:

AF	Amorphous metallic fiber
CNTs	Carbon nanotubes
CF	Carbon fiber
PEDOT	poly(3,4-ethylenedioxythiophene)
PSS	poly(styrenesulfonate)
